# Influence of cell specific productivity on product quality

**DOI:** 10.1186/1753-6561-5-S8-P95

**Published:** 2011-11-22

**Authors:** Ruchika Srivastava, Lavanya Rao, Kriti Shukla, Sunaina Prabhu, Saravanan Desan, Dinesh Baskar, Ankur Bhatnagar, Anuj Goel, Harish Iyer

**Affiliations:** 1 Cell Culture Lab, Biocon Limited, Bangalore, India

## Introduction

The micro heterogeneity or quality of a protein has been shown to have a significant impact on its physical, chemical and biological properties both in vitro and in vivo [1]. Micro heterogeneity is evaluated in terms of post translational modifications such as glycosylation, charge variants, aggregates and fragments profile. The biggest challenge in process development is to find a balance between increasing productivity while maintaining product quality.

In our study we focus on protein glycosylation, which is a process in which oligosaccharides are added to the protein during synthesis. There are multiple possible reactions in the pathway and it takes a long time for a glycosylated protein to be fully processed. If some protein molecules have a shorter residence time in the ER and Golgi, the glycan may be only at an intermediate stage [1]. For recombinant glycoprotein, increase in cell specific productivity (amount of product produced per cell per unit time) which may result in shorter residence time in the ER and Golgi, must be weighed against possible changes in product quality attributes like glycosylation [2]. Our study concludes that it is possible to produce a protein with desired product quality profile with high specific productivity. Two different clones with the same productivity can have different product quality profiles; alternatively, the same clone with different specific productivity can be manipulated to produce the same desired product quality by altering the cell culture parameters or addition of supplements. This observation also influences the acknowledged methodology for selecting clones with higher productivity while still maintaining their product quality profile. Various process manipulations were evaluated as an attempt to improve on the product quality profiles without compromising the productivity.

## Materials and methods

Three CHO cell lines (A, B & C) expressing three different Antibodies (Ab1, Ab2 & Ab3) were cultured in commercially available animal component free media in 125 ml Erlenmeyer shake flasks and BIOSTAT B-DCU lab bioreactors. Cell Count and Viability were analyzed by Cedex Hires (Innovatis) and heamocytometer using Trypan blue dye exclusion. The product concentration was determined by Affinity chromatography and characterization (Glycan profiling) by Normal phase HPLC.

## Results and discussion

### Clone Selection program

**Figure 1:**
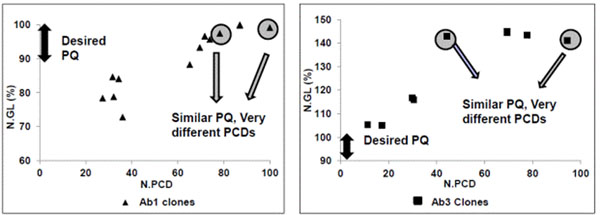
Plot of N.PCD vs. N.GL % for Ab1 & Ab3 suggest that there are some clones which have very different PCDs but similar product quality.

Figure 1 shows the plot of N.PCD (normalized specific productivity – picogram per cell per day) vs. N.GL % (normalized values of one type of glycosylated species) of different clones for the antibodies Ab1 & Ab3. Both show a similar general trend indicating an increase in N. GL (%) with increasing specific productivities. There are however some exceptions where clones with significantly different specific productivity show very similar glycosylation profile, which suggest the role of process conditions in affecting the product quality.

### Case study 1: Ab1

**Figure 2:**
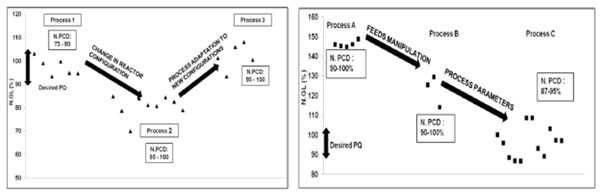
a: Profiles of Process 1, 2 & 3 for Ab1 Figure 2b: Profiles of Process A, B & C for Ab3

As seen in (Figure 2a), the desired N. GL (%) for Ab1 was comparable to the product obtained from the high PCD clones in Process 1. However, when the process was run in a different reactor configuration, a decrease in N.GL (%) was observed. Experiments were done to understand the impact of changes in the reactor conditions by varying the reactor dependent parameters (aeration, agitation etc) and the feeding strategy. These results were used to modify the Process 2 and made as a more robust Process 3. The Process 3 was able to give a higher value of N.GL (%) while still retaining the high PCD.

### Case study 2: Ab2

All the high producing clones for Ab2 were giving significantly higher N.GL (%) compared to the desired quality. A study was conducted to evaluate the possibility of choosing the high producing clone and manipulate the glycan profiles to be able to meet the product quality requirements. Intermittent samples were taken from the Fed batch runs and analyzed for product conc. and glycan profiles. Both PCD and N.GL (%) vary during the course of the run with a general trend of higher N.GL(%) with increase in PCD. However there were exceptions like day 8 vs. day 12 where the PCD of day 8 was significantly lower than day 12 however the N.GL(%) value was higher for the day 8. The feeding strategy and the process parameters (controlled and measured) around day 8 were estimated and compared to other days.

The results of the investigation showed that a feed added at around day 8 during the batch, helped in reducing the N. GL (%) values. The quantity of this feed as well as its feeding strategy were changed which helped in getting the desired product quality. The results from the study done above were implemented in the process.

### Case study 3: Ab3

The desired N. GL (%) for the product was significantly lower than the range obtained from most of the clones. The process used here is referred to as Process A. Feeding concepts implemented in the Ab2 improved process were tested in the Ab3 which is referred as Process B.The feed manipulations helped in bringing down the N.GL (%) values. However they were still much higher than the desired range. The time course analysis of the Process B batches was done. The analysis indicated some days during the run where the level of N.GL (%) were much lower compared to other days. Detailed analysis of the culture conditions during these days was done. The outcome of these analysis were implemented in the process by changing the culture parameters during the run. This process is referred as Process C. The profiles of batches run with Processes A, B and C are shown in (Figure 2b). Significant decrease in the level of N. GL (%) was observed in Process C without affecting the PCD of the cells.

## Conclusion

It was generally observed that the clones with high specific productivity also have high levels of N.GL (%). Since some of the products required lower levels of N.GL (%); these high producing clones were found to be unsuitable. However, the results of this study show that with modified conditions of process parameters and feeding strategies, it was possible to alter the N.GL (%) levels without impacting the cell line specific productivity. These factors when taken into consideration during clone selection may still allow the selection of high producing clones with a possibility to alter the product profiles during development.
